# Improved Approach for Chondrogenic Differentiation of Human Induced Pluripotent Stem Cells

**DOI:** 10.1007/s12015-014-9581-5

**Published:** 2015-01-13

**Authors:** Hossein Nejadnik, Sebastian Diecke, Olga D. Lenkov, Fanny Chapelin, Jessica Donig, Xinming Tong, Nikita Derugin, Ray C. F. Chan, Amitabh Gaur, Fan Yang, Joseph C. Wu, Heike E. Daldrup-Link

**Affiliations:** 1 Department of Radiology, and Molecular Imaging Program at Stanford (MIPS), Stanford School of Medicine, Stanford, CA 94304 USA; 2 Max Delbrück Center, Robert-Rössle Strasse 10, 13125 Berlin, Germany; 3 Berlin Institute of Health, Luisenstraße 56, 10117 Berlin, Germany; 4 Department of Orthopedic Surgery, Stanford School of Medicine, Stanford, CA 94305 USA; 5 BD Biosciences, Custom Technology Team, La Jolla, CA 92037 USA; 6 Department of Bioengineering, Stanford University, Stanford, CA 94305 USA; 7 Stanford Cardiovascular Institute, Stanford School of Medicine, Stanford, CA 94305 USA

**Keywords:** Pluripotent stem cell, Mesenchymal stem/stromal cell, Cartilage tissue engineering, MRI (magnetic resonance imaging), Osteoarthritis

## Abstract

Human induced pluripotent stem cells (hiPSCs) have demonstrated great potential for hyaline cartilage regeneration. However, current approaches for chondrogenic differentiation of hiPSCs are complicated and inefficient primarily due to intermediate embryoid body formation, which is required to generate endodermal, ectodermal, and mesodermal cell lineages. We report a new, straightforward and highly efficient approach for chondrogenic differentiation of hiPSCs, which avoids embryoid body formation. We differentiated hiPSCs directly into mesenchymal stem /stromal cells (MSC) and chondrocytes. hiPSC-MSC-derived chondrocytes showed significantly increased Col2A1, GAG, and SOX9 gene expression compared to hiPSC-MSCs. Following transplantation of hiPSC-MSC and hiPSC-MSC-derived chondrocytes into osteochondral defects of arthritic joints of athymic rats, magnetic resonance imaging studies showed gradual engraftment, and histological correlations demonstrated hyaline cartilage matrix production. Results present an efficient and clinically translatable approach for cartilage tissue regeneration via patient-derived hiPSCs, which could improve cartilage regeneration outcomes in arthritic joints.

## Introduction

Osteoarthritis (OA) is a major cause of disability, affecting about 43 million individuals in the US [1] and resulting in significant medical costs and lost wages reaching up to $95 billion per year [2]. Permanent articular cartilage defects, characterized by deterioration of the collagen matrix and depletion of aggrecan and type 2 collagen, represent the primary cause of OA [3], and are difficult to treat because cartilage cannot self-regenerate [4]. To address this problem, chondrocyte and bone marrow derived stem cell transplants have been explored as a therapeutic option for cartilage regeneration. However, both cell types are limited by several drawbacks, including an insufficient number of collectable donor cells, invasiveness of the harvesting procedure, and tendency of these cell types to form undesired fibrocartilage [5].

Pluripotent stem cells have demonstrated great potential for restoration of desired hyaline cartilage [6]. Recently, autologous human induced pluripotent stem cells (hiPSCs), generated from adipose-derived stem cells (ASCs) [7] or fibroblasts [8, 9] using virus independent reprogramming techniques, have been introduced as a clinically applicable source for creation of patient-specific cartilage [10, 11]. Unlike allogeneic cells, autologous hiPSCs do not engender immune reactions, and unlike embryonic stem cells, they do not raise ethical concerns [9, 12]. In addition, hiPSCs overcome limitations associated with autologous bone marrow-derived stem cells, such as invasive harvesting procedures, variable yields, and restricted cartilage regeneration potential of cells obtained from older patients [13].

**Fig. 1:**
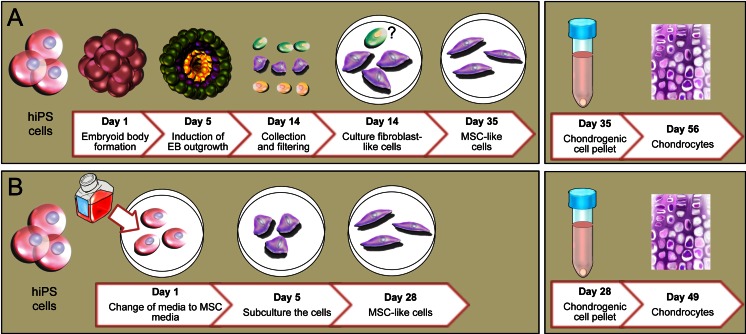
Chondrogenic differentiation of hiPSC. (**a**) Classical chondrogenic differentiation of hiPSCs via formation of embryoid bodies, outgrowth of endodermal (*green*), ectodermal (*yellow*) and mesodermal (*red*) cell lineages, selection of mesodermal cells, and induction of MSC and induction of chondrocytes. In this method hiPS cells were detached from matrigel coated dish and moved to ultra low attachment culture dish for 5 days to induce the EB formation, then EBs moved to plastic culture dish to select the hMSCs by collecting the outgrowing cells from EB (from day 5 to day 14) after collecting the attached fibroblast-like cells. These cells were cultured for 3 weeks in media containing FBS to prepare the hiPSC-MSCs (day 35 of differentiation). Then, hiPSC-MSCs were differentiated in a pellet culture system using serum free chondrogenic media for 3 weeks. (**b**) Embryoid body free method of direct differentiation of hiPSCs into hiPSC-MSCs, followed by chondrogenic differentiation. In embryoid body free method hiPSCs were cultured in matrigel coated dish and media was changed to hMSC media (DMEM supplemented with FBS) for 5 days to induce the hMSC differentiation (Day 5). Then, cells were detached and moved to a plastic culture dish for 4 passages to prepare the hiPSC-MSCs (Day 28). To differentiate the hiPSC-MSCs to chondrocytes, cells were used in pellet culture system using serum free chondrogenic media for 3 weeks

While hiPSCs have shown promise for cartilage defect repair, the complex and inefficient process used to differentiate hiPSCs to cartilage limits the clinical translation of this approach [14]. The most frequently used technique requires three main steps: (1) formation of suspension embryoid bodies; (2) mesenchymal stem/stromal cell (MSC) outgrowth from embryoid bodies; and (3) selection of MSC via cell sorting and induction of chondrogenic differentiation [14] [15], (Fig. 1). This approach is highly inefficient, as it leads to a variable number and size of embryoid bodies, which are composed of heterogeneous cell populations, and results in unpredictable differentiation to undesired cell lines [16]. We hypothesized that eliminating embryoid body formation as an intermediate step in the differentiation process could reduce generation of unwanted cell lines and improve the yield of chondrocytes.

Thus, the goal of our study was to develop a novel approach for differentiation of hiPSCs that sidesteps embryoid body formation and improves the efficiency of chondrocyte production from hiPSCs. In contrast to prior work on chondrocyte differentiation, our approach involves direct induction of human mesenchymal stem/stromal cells (hMSCs) under specific cell culture conditions, followed by classic chondrogenic differentiation (Fig. 1). Our approach is superior over its predecessors because it bypasses differentiation into undesired cell types. This could be exploited as a framework for more efficient and better controlled design of new cellular therapeutics for cartilage regeneration. Our technique could be also widely applied to other pluripotent stem cells (e.g., ESCs) and other differentiation pathways, beyond our own focus of cartilage regeneration.

## Materials and Methods

### hiPSC Culture and Pluripotency Evaluation

**Fig. 2:**
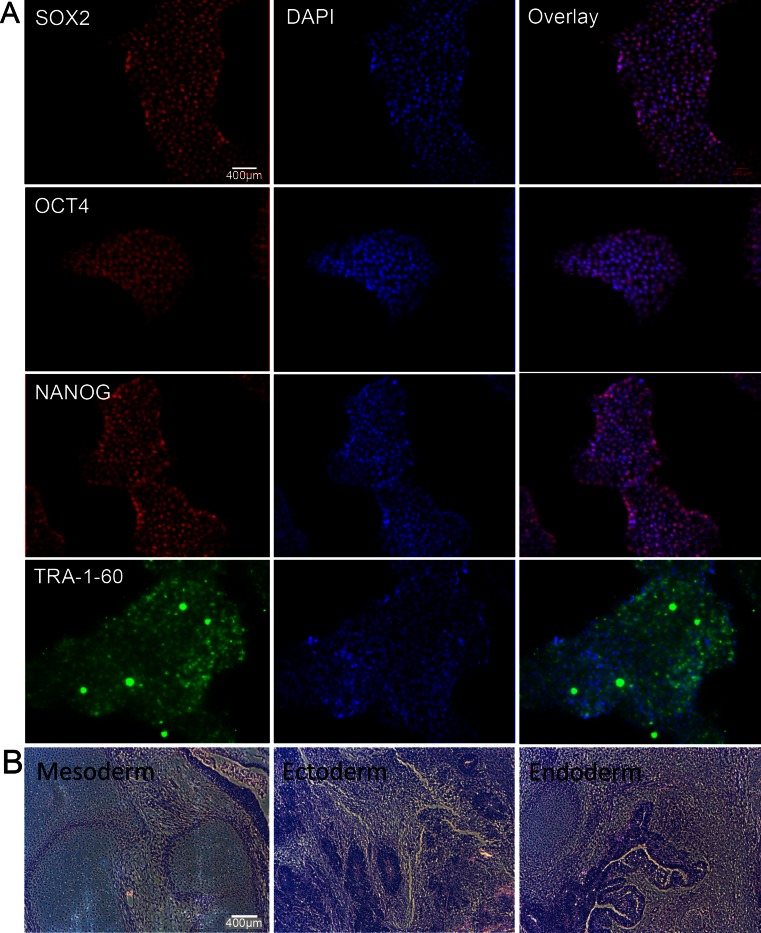
Pluripotency evaluation and teratoma formation of hiPSCs. (**a**) Immunofluorescence staining with DAPI counterstain demonstrating positive pluripotency markers NANOG, OCT4, SOX2, and TRA-1-60. (**b**) H&E stains of a representative hiPSC-derived teratoma confirm pluripotency of the hiPSCs with presence of all three germ layers, including ectoderm, mesoderm, and endoderm. (scale bar = 400 μm)

The study was approved by the Committee on Human Research and the Stem Cell Research Oversight (SCRO) Committee at our institution. Integration-free hiPSCs were derived from either adipose-derived stem cells (ACSs) using the previously described minicircle reprogramming technique, or from fibroblasts using a new optimized minicircle backbone [7]. hiPSCs derived from two different sources were used to proof that our iPS-MSC differentiation method can be applied to different hiPS cell types. In brief, to induce pluripotency we transfected (electroporation) 12 μg of codon optimized minicircle plasmids into 1 × 10^6^ adult human fibroblasts and plated the cells on Matrigel coated plates in DMEM 10 % FBS. On the following day, we changed the media with DMEM 10 % FBS plus 10 μm sodium butyrate and ascorbic acid (50 μg/ml). After 5 days we changed to chemical defined media E6 plus FGF2 (100 ng/ml). The first hiPSC colonies appeared after 30 days and were picked individually. Thereafter the undifferentiated hiPSCs were cultured in chemical defined conditions either in mTeSR1^™^ medium (Stem Cell^™^ Technologies, Vancouver, BC, Canada) or E8 (Life Technologies, Carlsbad, CA, USA) in 10 cm petri dishes (BD Falcon, Sparks, MD, USA) coated with 1 % matrigel (BD Matrigel™ Basement Membrane Matrix) at 37 ° C in a 5 % CO_2_ atmosphere. The medium was changed every day and cells were sub-cultured every 4–5 days using Accutase. Pluripotency of the hiPSCs were evaluated by immune-staining for pluripotency markers OCT4, SOX2, NANOG, and TRA-1-60 (Fig. 2a). A subcutaneous injection of hiPSCs was performed in severe combined immunodeficient (SCID) mice to evaluate the pluripotency phenotype of the hiPSCs. The resulting teratomas were sectioned and stained to verify all three germ layers’ differentiation, including endoderm, mesoderm, and ectoderm (Fig. 2b).

### hiPSC Differentiation into hiPSC MSC Cells

**Fig. 3:**
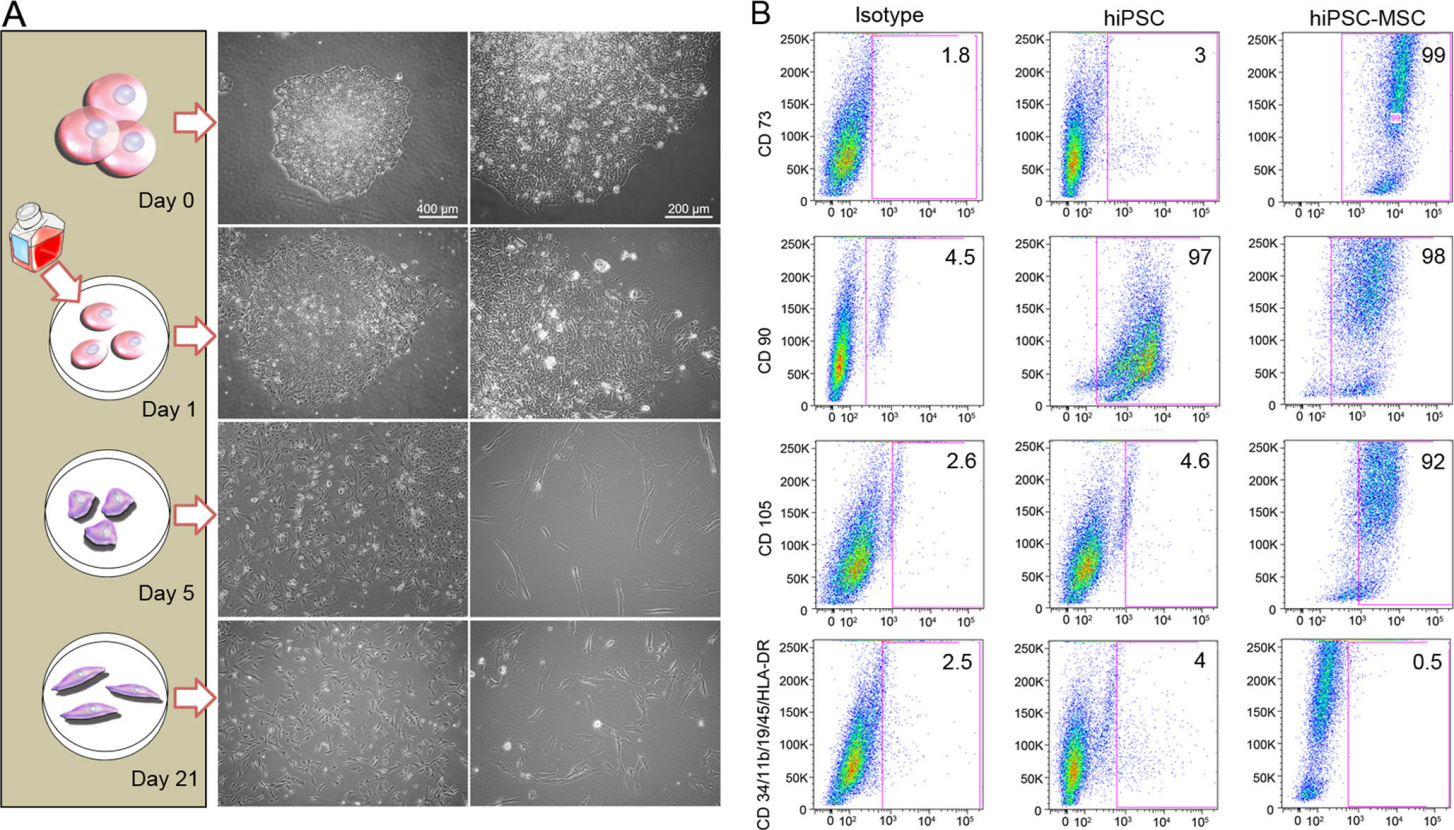
Morphology and Phenotypes of hiPSC-derived hiPSC-MSC cells. (**a**) Changes in cell morphology during hiPSC differentiation: Day 0: Dome-shaped hiPSC colony; Day 1: Changing the mTeSR1^™^/E8 medium to hMSC medium leads to differentiation and out-growth of the cells from the colonies; Day 5: After sub-culture to uncoated and untreated culture flasks, the pre-differentiated cells attach to the polystyrene flask and start to form an elongated morphology. Day 21: At passage 4, cells show a spindle shape morphology, similar to hMSCs. (scale bar left panel = 400 and right panel 200 μm). (**b**) Flow cytometry analysis of surface markers of hiPSCs and hiPSC-MSCs at passage 4 shows positive hMSC surface markers of CD105, CD73, and CD90, and lack of CD45, CD34, CD14 or CD11b, CD19 and HLA-DR surface molecules according to the International Society for Cell Therapy (ISCT) criteria

In vitro experiments were performed for both ASC-derived and fibroblast-derived hiPSCs. hiPSCs were differentiated into MSCs as shown in Fig. 2; Undifferentiated hiPSCs were cultured to reach 50 % confluency; Subsequently, the mTeSR1^™^/E8 media was changed to typical hMSC culture medium such as high glucose Dulbecco’s Modified Eagle Medium (DMEM, Life Technologies, Carlsbad, CA, USA) with 10 % stem cell-qualified fetal bovine serum (FBS, Life Technologies, Carlsbad, CA, USA), 100 units/mL of Penicillin, and 100 mg/mL of Streptomycin (Life Technologies, Carlsbad, CA, USA). The DMEM high-glucose medium was changed every day for 5 days. On day 5, cells were detached from the matrigel-coated petri dishes using 5 % Trypsin/EDTA (Life Technologies, Carlsbad, CA, USA) and cultured on uncoated polysterene culture flasks (Fisher Scientific Company, Pittsburgh, PA, USA). The medium was changed every other day until the cells reached 90 % confluency. The cells were then sub-cultured at a ratio of 1:3 until passage 4 (P4). The cell morphology of original hiPSCs, hiPSC-MSCs, and bone marrow derived MSCs were observed by phase contrast imaging over time (Fig. 3a).

### Phenotyping of hiPSC-MSC Cells

In order to characterize the phenotypes of the differentiated hiPSC-MSCs, triplicate samples of hiPSCs and hiPSC-MSCs underwent flow cytometry analyses on a BD FACS Canto II flow cytometer. Compensation was set using BD Comp Beads. The cells were tested for MSC markers according to the International Society for Cell Therapy (ISCT) [17] criteria, which included the presence of CD105, CD73, and CD90, as well as the lack of CD45, CD34, CD14 or CD11b, CD19 and HLA-DR surface molecules. Briefly, cells were stained using hMSC Analysis kit (BD Biosciences, CA) according to manufacturer instruction. Data were analyzed using BD FACS Diva software (BD Biosciences, San Jose, CA, USA) and Flowjo™ data analysis package (http://www.Treestar.com). Pluripotency markers of the hiPSC-MSCs were evaluated by immune-staining for pluripotency markers OCT4, SOX2, NANOG, and TRA-1-60.

### Chondrogenic Differentiation of hiPSC in vitro

To evaluate the chondrogenic potential of the hiPSC-derived MSCs in vitro, the cells were detached from culture flasks using 5 % Trypsin/EDTA and underwent chondrogenic differentiation in a 3D, high-density pellet culture using established protocols [18]. In brief, centrifuged pellets of 2.5 × 10^5^ hiPSC-derived MSCs were incubated in 5 % CO_2_ at 37 ° C and in 0.5 mL of serum-free chondrogenic differentiation medium, consisting of high glucose DMEM, 100 U/mL penicillin, 100 μg/mL streptomycin, 10 % L-Glutamine (Gibco), 50 μg/mL L-ascorbic acid 2-phosphate sequimagnesium (Sigma), 100 μg/mL MEM sodium pyruvate (Gibco), 40 μg/mL L-Proline (Sigma), 100 nM dexamethasone (Sigma), ITS + Premix final concentration: 5.5 μg/mL transferring, 10 μg/mL bovine insulin, 5 μg/mL sodium selenite, 4.7 μg/mL linoleic acid, and 500 μg/mL bovine serum albumin (BD Bioscience, Franklin Lakes, NJ), and supplemented with 10 ng/mL TGF-β3 (R&D Systems, Minneapolis, MN). The chondrogenic medium was changed every other day for 21 days. Pellets were harvested on days 0, 7, and 14 of chondrogenic differentiation for gene expression analysis and day 21 for standard histopathology and immunohistochemistry.

Quantitative real-time PCR (qPCR) was used to further confirm chondrogenic differentiation of the hiPSC-derived MSCs. Gene expression levels of the differentiated cells were assessed for hyaline cartilage markers (collagen type II (Col2A1), collagen type IX, collagen type XI, SRY (sex determining region Y)-box 9 (SOX9), and Aggrecan (ACAN)), fibrocartilage marker (collagen type I), hypertrophic cartilage (collagen type X), and the control marker of Glyceraldehyde 3-phosphate dehydrogenase (GAPDH). The primer sequences presented in Table 1. In brief, the total cellular RNA was extracted from each sample with the QIAGEN RNeasy® mini kit. Samples of cDNA were prepared from total RNA samples and qPCR was carried out on an Applied Biosystems StepOne™ Real-Time PCR System. The formation of double-stranded DNA was monitored by TaqMan® gene expression primers. Expression data were collected as Ct values and the gene expression levels were normalized to the reference control gene, GAPDH.

**Table 1:** Reference and target gene primer sequences used in qPCR experiments

Gene name	Sequence (F: Forward / R: Reverse)	Catalogue number	Reference
GAPDH*	F: 5′ CGCTCTCTGCTCCTCCTGTT 3′ R: 5′ CCATGGTGTCTGAGCGATGT 3′	Hs02758991_g1	NM_001256799
ACAN	F: 5′ AGGCAGCGTGATCCTTACC 3′ R: 5′ GGCCTCTCCAGTCTCATTCTC 3′	Hs00153936_m1	NM_001135
SOX9	F: 5′ GTACCCGCACTTGCACAAC 3′ R: 5′ TCTCGCTCTCGTTCAGAAGTC 3′	Hs01001343_g1	NM_000346
COL2A1	F: 5′ CGTCCAGATGACCTTCCTACG 3′ R: 5′ TGAGCAGGGCCTTCTTGAG 3′	Hs00264051_m1	NM_001844
COL1A2	F: 5′ CAGGAAACAGCTATGACC 3′ R: 5′ CTACTCTCAGCCCAGGAGGTCCTG 3′	Hs01028969_m1	NM_000089
COL9A1	F: 5′ TGTAAAACGACGGCCAGT 3′ R: 5′ CAGGAAACAGCTATGACC 3′	Hs00932129_m1	NM_001851
COL10A1	F: 5′ GGCAGAGGAAGCTTCAGAAA 3′ R: 5′ AAGGGTATTTGTGGCAGCATA 3′	Hs00166657_m1	NM_000493
COL11A1	F: 5′ TGTAAAACGACGGCCAGT 3′ R: 5′ CAGGAAACAGCTATGACC 3′	Hs01097664_m1	NM_001190709

To investigate the Aggrecan and Collagen type II production of the cells, Alcian blue and immunohistochemistry staining were performed. The chondrogenic pellets were fixed in 10 % neutral buffered formalin (VWR, PA, USA), dehydrated through graded alcohol washes (70, 95 and 100 %) and xylene (EMD, Millipore, USA), embedded in paraffin and sectioned into 5 μm thick tissue slices on glass slides. The slides were de-waxed to undergo Hematoxylin and Eosin (H&E) staining for evaluation of cell morphology, Alcian blue staining for detection of proteoglycan production, and immunohistochemistry for Collagen type II detection. For Collagen type II immunohistochemistry, tissue sections were pre-digested with pepsin (1 mg/mL in Tris–HCl, pH 2.0), incubated with the anti-collagen II primary antibody (Chemicon, 1:500) for 60 min, followed by biotinylated goat anti-mouse antibody for 30 min and streptavidin peroxidase for 45 min at room temperature. Sections were visualized with DAB chromogen, counterstained with Hematoxylin for 3 min, dehydrated, and mounted with Permount solution.

### Osteogenic and Adipogenic Differentiation of hiPSC in vitro

To evaluate the osteogenic and adipogenic potential of the hiPSC-MSCs in vitro, the cells were detached from culture flasks using 5 % Trypsin/EDTA and underwent differentiation. Osteogenic differentiation was induced by culturing 6 × 10^4^ cells/cm^2^ in osteogenic differentiation medium consisting of DMEM supplemented by 10 % FBS (Gibco), 100 U/ml penicillin, 100 μg/ml streptomycin (Gibco), 10 % L-Glutamine (Gibco), 50 μg/ml L-ascorbic acid 2-phosphate sequimagnesium (Sigma), 100 μg/ml MEM sodium pyruvate (Gibco), 0.1 μM dexamethasone (Sigma), and 100 mM b-glycerophosphate. Adipogenic differentiation was induced by culturing 3 × 10^5^ cells/cm^2^ in adipogenic medium consisting of DMEM supplemented with 10 % FBS, 100 U/ml penicillin, 100 μg/ml streptomycin (Gibco), 10 % glutamax (Gibco), 100 μg/ml insulin (Sigma), 500 μM 3-isobuthy-l-methylxanthine (IBMX), 100 μM indomethacin and 1 μM dexamethasone (Sigma). The medium was changed every 3–4 days for 3 weeks.

Histological evaluation of osteogenic differentiation was evaluated using Alizarin Red S stain. Cells were stained with the 2 % Alizarin Red S solution (pH 4.1 ~4.3) for 5 min at room temperature, and the reaction were observed microscopically. Cells were washed with distilled water to remove the excess stains. Calcium deposits in differentiated cells would produce red-orange stains.

Histological evaluation of adipogenic differentiation was determined using 0.3 % Oil Red O stain for 15 min at room temperature to stain intracellular lipids, and counterstained with hematoxylin. Fat vacuoles in differentiated cells would produce red stains.

### Engraftment of hiPSC-derived MSCs and Chondrogenic Pellets in rat Knee Joints

In vivo experiments were performed with hiPSC-MSC differentiated from ASC derived hiPSC only. To evaluate in vivo engraftment and exclude in vivo teratoma formations, hiPSC-MSCs and chondrocytes were implanted into osteochondral defects of rat knee joints and evaluated with MR imaging and histopathology. The animal experiments were approved by the animal care and use committee at our institution. Osteochondral defects were created in the distal femoral trochlear groove of 9 knee joints of 5 athymic nude Sprague Dawley rats, using a micro-drill (Flash DP Tabletop Micromotor, DBI America Corp, FL, USA). 2.5 × 10^5^ hiPSC-derived MSCs (3 knees) or 2.5 × 10^5^ hiPSC-derived chondrogenic differentiated cell pellets after 3 weeks of differentiation (3 knees) in 2 μl of Polyethylene Glycol (PEG) and chondroitin sulfate methacrylate (CS) based scaffold were implanted into the femoral defect. Scaffold-only implants (3 knees) served as controls.

The PEG-CS scaffold was prepared afresh every time before the cell implantation by mixing 14 μl of 10 % PEG3K-DMA solution with 6 μl of 10 % CS solution. Directly before implantation, cells in PEG-CS were mixed with 2.4 μl of polymerizing solution (containing two parts of Ammonium Persulfate (APS) solution (1 M) and one part of tetramethylethylenediamine (TMEDA) solution (1 M)), and immediately injected to the defect. The cell-seeded scaffold polymerized within 2 min.

To exclude teratoma formation and investigate the engraftment of cell implants over time, all knee joints underwent MR imaging immediately following stem cell transplantation as well as 3 weeks and 6 weeks after transplantation. MR imaging was performed on a 7 Tesla MR scanner (General Electric “microSigna 7.0”) using a single-channel transmit/receive partial birdcage radiofrequency coil. Sagittal MR images of the rat knees were obtained with a fast spin echo (FSE) sequence (Repetition time, TR: 3000 ms, Echo time, TE: 30 ms) and a multi-echo spin echo (SE) sequence (TR 4000 ms /TE 15, 30, 45, 60 ms), using a field-of-view (FOV) of 2.5 × 2.5 cm, a matrix of 256 × 256 pixels, and a slice thickness of 0.5 mm. Because successful engraftment has been characterized by a significant decline in T2-relaxation times of cell implants in cartilage defects [19], we generated pixel-wise T2 relaxation time maps of cell implants using custom research software (Cinetool, GE Global Research Center, Niskayuna, NY). T2 relaxation times of each cell implant was measured on these maps via operator-defined regions of interests (ROIs). After the last MR scan, animals were sacrificed and specimens were processed for postmortem histopathology correlations, which included H&E stains, immunohistochemistry for collagen type II, and Alcian blue stains. ICRS (International Cartilage Repair Society) visual histological assessment scale was used as a standard histological grading to quantify the extent of cartilage repair (Table 2).

**Table 2:** ICRS visual histological assessment scale

Feature	Score
I. Surface	
Smooth/continuous	3
Discontinuities/irregularities	0
II. Matrix	
Hyaline	3
Mixture: hyaline/fibrocartilage	2
Fibrocartilage	1
Fibrous tissue	0
III. Cell distribution	
Columnar	3
Mixed/columnar-clusters	2
Clusters	1
Individual cells/disorganized	0
IV. Cell population viability	
Predominantly viable	3
Partially viable	1
<10 % viable	0
V. Subchondral Bone	
Normal	3
Increased remodeling	2
Bone necrosis/granulation tissue	1
Detached/fracture/callus at base	0
VI. Cartilage mineralization (calcified cartilage)	
Normal	3
Abnormal/inappropriate location	0

Engraftment and long term viability of the implanted cells evaluated by human anti-nuclear specific immunofluorescent stain (MAB1281 | Anti-Nuclei Antibody, EmdMillipore) that was performed on the rat knee samples.

### Data Analysis

Gene expression levels of original hiPSCs, intermediate hiPSC-derived MSCs as well as chondrogenic pellets at days 0 and 14 of chondrogenic differentiation were compared using an analysis of variance (ANOVA). A Bonferroni correction was applied for comparisons. An analysis of variance (ANOVA) with a Bonferroni correction was used to compare MRI T2 relaxation times of each groups overtime, and the T2 relaxation times of the scaffold-only group were compared with hiPSC-derived MSC and hiPSC chondrogenic differentiated cell pellets groups. All statistical analyses were performed using GraphPad Prism 6 statistical software (GraphPad Software Inc. CA, USA).

## Results

### Generation of hiPSC-MSCs from hiPSCs Without Intermediate Embryoid Body Formation

In order to enable clinical use, it is important to use hiPSC-derived cell products without viral integrations. Therefore, we generated hiPS cell lines from human adipose derived stem cells, using the minicircle reprogramming technique [7]. For generation of hiPS cell lines from adult human fibroblasts, we used a codon-optimized minicircle plasmid [20] (Diecke et al. 2014). The pluripotency of the generated hiPSCs was confirmed by positive immunofluorescence staining for NANOG, OCT4, SOX2, and TRA-1-60 (pluripotency markers) (Fig. 2a). In addition, H&E stains of hiPSC-derived teratomas showed differentiation into all three germ layers, including ectoderm, mesoderm, and endoderm (Fig. 2b).

To initiate hMSC-induction, undifferentiated hiPSCs were cultured in DMEM high-glucose medium for 5 days, followed by transfer to uncoated polysterene culture flasks, continued culture until 90 % confluency and sub-culture at a ratio of 1:3 until passage 4 (P4).

The change of mTeSR1^™^/E8 medium to hMSC medium resulted in a change in cell morphology from dome-shaped hiPSC colonies to elongated and spindle-shaped cells, which spread out from their original colonies. Although the cells demonstrated a heterogeneous morphology on the first and second day of MSC-induction, more than 90 % of the total cell population acquired a fibroblast-like morphology by passage 4, matching the typical morphology of hMSCs (Fig. 3a). To determine phenotypes and genotypes of hiPSC-derived MSCs, we performed standard microscopy and flow cytometry analysis. The flow cytometry analysis showed that more than 90 % of the cells were positive for the hMSC markers: CD105 (>91 %), CD73 (>96 %), and CD90 (>95 %), and negative for CD45, CD34, CD14 or CD11b, CD19, and HLA-DR surface molecules (>95 %) (Fig. 3b). The results showed that hiPSCs are positive for CD90 which is in accordance to other researcher that suggested CD90 as one of the pluripotency markers [21]. The differentiation of the generated hiPSC-MSCs was confirmed by negative immunofluorescence staining for pluripotency markers such as NANOG, OCT4, SOX2, and TRA-1-60 (data not shown).

**Fig. 4:**
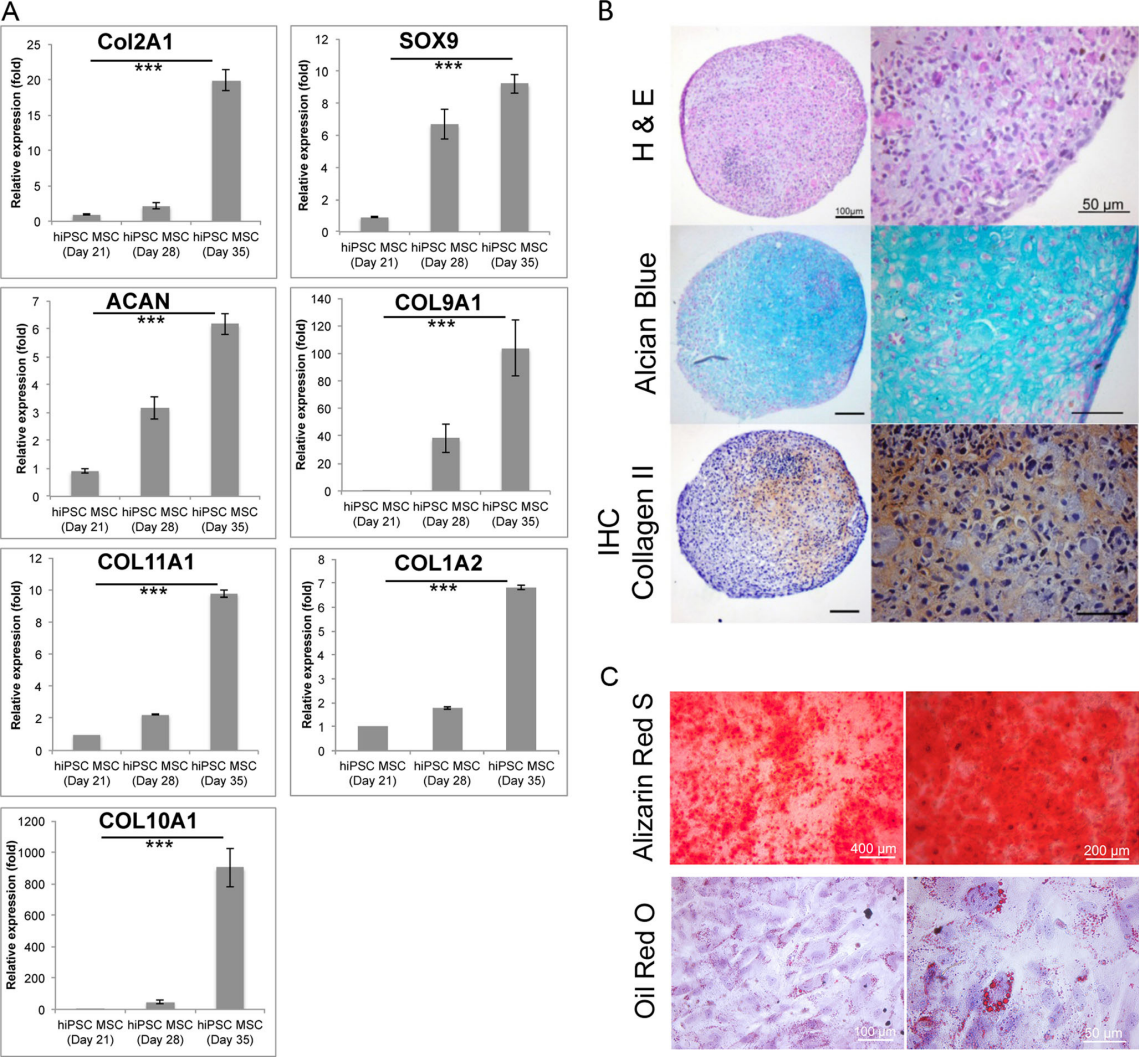
Characterization of hiPSC-derived Chondrogenic Cells. (**a**) Relative gene expression of hiPSC-derived hiPSC-MSCs at day 21 (equal to day 0 of chondrogenic differentiation) and chondrogenic cell pellets at day 28 (equal to day 7 of chondrogenic differentiation) and 35 (equal to day 14 of chondrogenic differentiation), as determined by qPCR. Data are displayed as means and standard errors of triplicate experiments per sample. Cells at day 14 of chondrogenic differentiation show significantly increased gene expression of the hyaline chondrogenic markers COL2A1, COL9A1, COL11A1, SOX9, and aggrecan (ACAN) compared to hiPSC-MSCs. They also show an increased expression of COL1A2 and COL10A1 representative of fibro- and hypertrophic cartilage respectively. (*** indicates *p* < 0.001). (**b**) Histological evaluation of hiPSC-derived chondrogenic cell pellets at day 42 (equal to day 21 of chondrogenic differentiation); H&E stain shows chondrocytes and formation of a chondrogenic matrix. Alcian blue stain demonstrates positive glycosaminoglycan production, and immunohistochemistry shows positive stains for Collagen type II. (left 100 μm, right 50 μm). (**c**) Histological evaluation of hiPSC-MSCs osteogenic (upper panel) and adipogenic (lower panel) differentiation. Alizarin Red S staining used for osteogenic differentiation evaluation and Oil Red O staining used to assess the adipogenic differentiation of hiPSC-MSCs after 3 weeks of differentiation

Alizarin Red S staining of the hiPSC-MSCs visualized the calcium deposition of the cells after 21 days of osteogenic differentiation induction which proves the osteogenic differentiation potential of the hiPSC-derived MSCs. In addition, Oil red O stain demonstrated that hiPSC- derived MSCs exhibited developing of fat vacuoles after 21 days of adipogenic differentiation (Fig. 4c).

### Chondrogenic Differentiation of hiPSC-derived MSCs

To evaluate chondrogenic differentiation of hiPSC-derived MSCs in vitro, we induced chondrogenic differentiation in a 3D, high-density pellet culture [18] and tested original hiPSCs, hiPSC-derived MSCs and chondrogenic pellets for cartilage markers. Col2A1, Col9A1, Col11A1, SOX9, and ACAN genes were significantly increased in chondrogenic cell pellets at day 14 of differentiation, confirming the differentiation of the cells towards chondrogenic lineage (*p* < 0.001) (Fig. 4a). However, additional upregulation of Co1A2 and Col10A1 indicates some components of fibrocartilage and hypertrophic cartilage, respectively (Fig. 4a). The histologic evaluation of the cell pellets at day 21 of chondrogenic differentiation (day 42 on Fig. 1) confirmed cartilage tissue formation on H&E stains and positive Alcian blue stains, indicating proteoglycan production (Fig. 4b). In addition, collagen type II immunohistochemistry was positive for chondrogenic pellets at day 21, indicating production of the hyaline cartilage matrix (Fig. 4b).

**Fig. 5:**
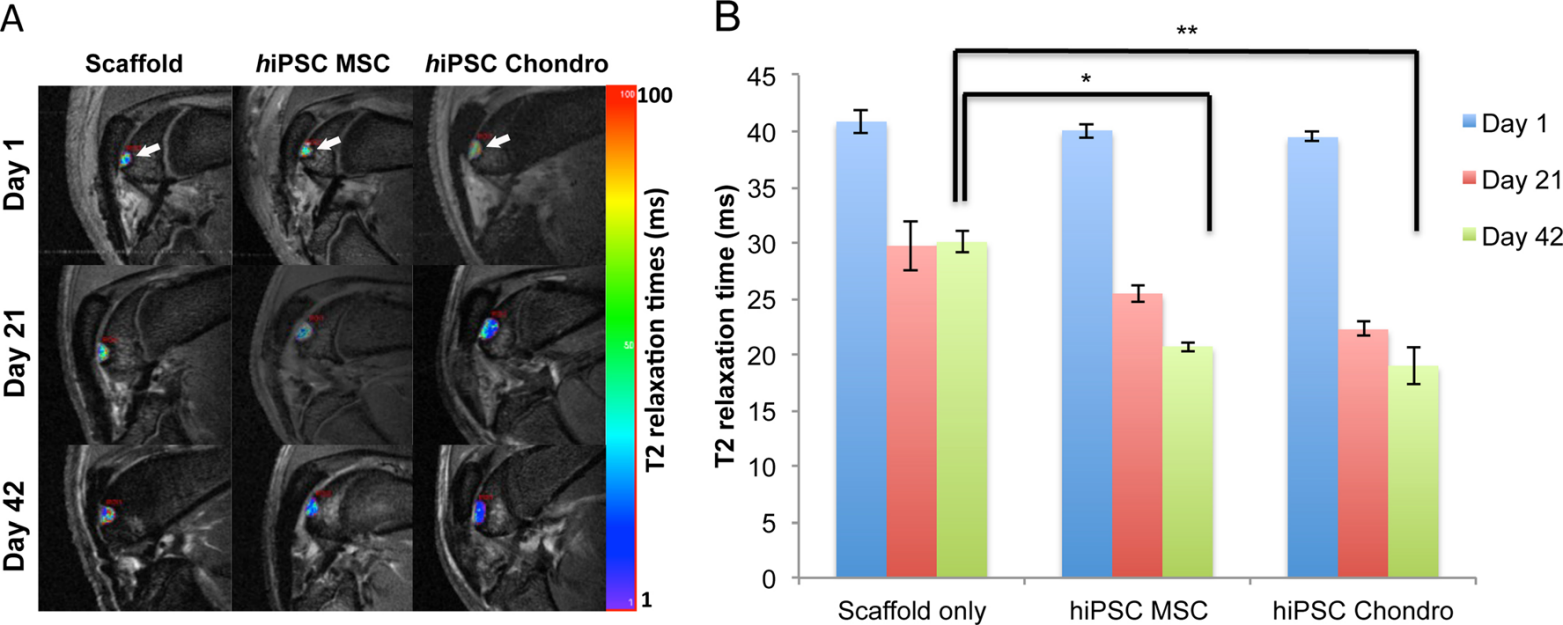
In vivo engraftment of hiPSC-derived MSCs and Chondrogenic Cells. (**a**) Sagittal T2-weighted MR images of implants of scaffold only, hiPSC-derived MSCs, and hiPSC-derived chondrogenic cells in osteochondral defects of the distal femurs of rat knee joints. Superimposed T2 relaxation time maps show decreasing T2 values of transplanted cells, but not scaffold only, over time. (**b**) Corresponding quantitative measures of T2-relaxation times of cell transplants and scaffold only. Data are displayed as means and SE of triplicate experiments. (* and ** indicates *p* < 0.05 and *p* < 0.01 respectively)

**Fig. 6:**
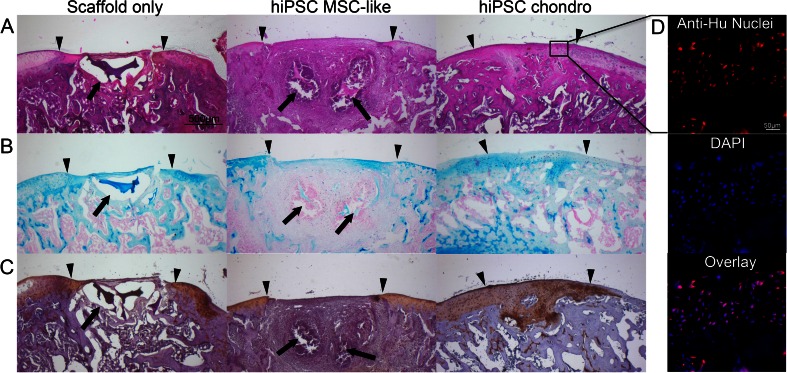
Histological evaluation of hiPSC-derived MSCs and chondrogenic cells implanted in rat knee joints. (**a**) H&E stain of chondrogenic differentiated hiPSC-derived MSCs shows persistent defect after transplantation of scaffold only and engraftment of cell implants with defect remodeling. (**b**) Alcian blue stain demonstrates no glycosaminoglycan (GAG) production of scaffold only, mildly positive GAG production of hiPSC-derived MSCs and markedly positive GAG production of chondrogenic cells. (**c**) Collagen II immunohistochemistry shows no production of Collagen type II in scaffold only and MSC transplants, but markedly positive Collagen type II production after transplantation of chondrogenic cells. (Arrow heads display the borders of the defect and the complete arrows show the residual of scaffold; scale bar is equal to 500 μm). (**d**) Human anti-nuclear specific immunofluorescent stain shows presence and long term viability of the human cells in the repaired tissue (Scale bar is equal to 50 μm)

In order to exclude teratoma formation and confirm chondrogenic differentiation of hiPSC-derived MSCs in vivo, hiPSC-derived MSCs and hiPSC-derived chondrogenic cells (day 21) were implanted into osteochondral defects of the distal femur of nude athymic Sprague Dawley rats and evaluated with serial MR imaging studies over a time period of 6 weeks. The MR imaging evaluation did not show any evidence for teratoma formation of hiPSC-derived MSCs or chondrogenic cells in vivo. The cell transplants showed significantly decreasing T2 relaxation times over time (*p* < 0.001). This signal effect is an indication for decreasing water content and increasing matrix formation over time and has been associated with successful engraftment as described by Trattnig et al. [19]. T2-relaxation times of cell implants at 6 weeks post implantation were significantly lower compared to controls with scaffold only implants (*p* ≤ 0.013) (Fig. 5). This corresponded on histopathology to higher cellularity and degradation of the scaffold in the cell transplants compared to scaffold only (Fig. 6, Table 3). H&E staining confirmed the engraftment of hiPSC-derived MSC cells and hiPSC-derived chondrogenic differentiated cell pellets in osteochondral defects. hiPSC-derived MSC implants had started to remodel the defect and to produce a chondrogenic matrix, as evidenced by positive Alcian blue stains and positive immunostains for collagen type II (Fig. 6). By comparison, scaffold-only implants demonstrated no repair of the defect. hiPSC-derived chondrogenic pellets showed stronger GAG and collagen type II staining compared to hiPSC-derived MSCs implants (Fig. 6). ICRS histological scale assessment revealed that the overall histological score of repaired cartilage were significantly higher in hiPSC-chondrocytes comparing to hiPSC-MSC implants (*p* < 0.032). Moreover, matrix production of the hiPSC-chondrocytes was significantly higher than hiPSC-MSC implants (*p* < 0.016).

**Table 3:** Histological results of cartilage repair of in vivo implants

	Scaffold only	hiPS-MSC	hiPSC-Chondrocyte
Surface	1 (1.73)	1 (1.73)	3 (0)
Matrix	0.34 (0.57)	1 (0)	2.34 (0.57)
Cell distribution	0.34 (0.57)	0.67 (0.57)	1.67 (0.57)
Cell population viability	0 (0)	3 (0)	2.34 (1.15)
Subchondral Bone	1.34 (0.57)	1.67 (0.57)	2 (0)
Cartilage mineralization	0 (0)	3 (0)	3 (0)
Overall	3 (1.73)	10.34 (0.57)	14.34 (2.08)

Data presented as mean score with standard deviation (SD)

Human anti-nuclear specific immunofluorescent stain confirmed the presence and long-term viability of the human cells in the repaired cartilage tissue in the hiPSC-derived chondrogenic cells (Fig. 6d).

## Discussion

Our findings show that a new and simplified differentiation protocol can be used to differentiate hiPSCs directly into mesenchymal stromal cells, without intermediate embryoid body formation, thereby providing a more efficient approach for cartilage generation from hiPSC compared to standard procedures. Our procedure yielded 90 % chondrocytes and successfully repaired osteochondral defects in vivo.

hiPSCs offer several advantages for cartilage repair over bone marrow-derived MSCs and chondrocytes [22]. Whereas chondrocytes and most adult stem cells such as hMSC and adipose derived stem cells (ADSCs) show decreasing proliferation and differentiation potential after 4 passages in culture [23], while undifferentiated hiPSCs can be expanded indefinitely. This allows for the generation of a high quantity of differentiated progeny for high-throughput analysis before their transplantation into patients [24–26]. Embryonic stem cells are the only other cell type with a similar capacity for proliferation. However, the allogeneic nature of embryonic stem cells may generate immune reactions, which limits their potential for clinical applications [24]. By contrast, hiPSCs from minimally invasive sources such as skin fibroblasts or fat cells allow for generation of autologous engineered tissues, even from elderly patients typically suffering from OA [27, 28].

Despite their potential advantages, the use of hiPSCs for cartilage repair has been limited because of the inefficiency and complexity of standard chondrogenic differentiation processes. Standard protocols require co-culture of hiPSCs with chondrocytes [29], which requires a surgery for chondrocyte harvest, as well as culture of hiPSCs in complex differentiation media, which contain multiple growth factors with unclear in vivo effects [30, 31]. In addition, the standard hiPSC differentiation approach via embryoid body formation gives rise to heterogeneous cell populations and unpredictable differentiation outcome [32]. Mesenchymal lineage selection from embryoid bodies is laborious and costly, providing comparatively low yields [33, 34].

Other investigators explored approaches for hiPSC differentiation without embryoid body formation by culturing hiPSCs as micromass formation (Saitta et al., 2014) [35], under pellet culture conditions (Phillips et al., 2014) [36], and using specific media on modified/coated culture flasks and/or on feeder-layers [37]. These methods may impede clinical translations by interfering with biomechanical properties of the substrate, suppressing cellular proliferation [38, 39], negatively affecting related signaling pathways [40, 41], using viral vectors to produce iPS cells, risk of mixing feeder layer cells with final cells [36], or increasing costs compared to standard techniques [42, 43]. Chen et al. reported that hiPSC incubation with SB431542 (a specific inhibitor of TGF-β receptor kinase) in two-dimensional cultures enabled MSC conversion without embryoid body formation [44]. However, their method required an intermediate step of epithelial differentiation and the clinical safety and efficacy of SB431542 are not yet established. By comparison, our method does not require the use of a feeder-layer or coating, does not involve either embryoid body formation or an endothelial conversion step, and requires no additional chemicals to differentiate the cells towards mesenchymal lineage.

We recognize several limitations of our study. Firstly, we observed some components of fibrocartilage and hypertrophic cartilage in our in vitro differentiation samples, which needs to be improved for future use by optimizing the in vitro chondrogenic differentiation induction method (e.g. by using different combination of growth factors).. We also used fetal bovine serum (FBS) as a supplement for cell culture, which may increase the risk of cell transformation and prion diseases. Substitution with fully defined serum-free media as described by Yamasaki et al. will increase the safety of our method for clinical applications [45]. Although we did not observe teratoma formations in our cell transplants, hiPSCs and hiPSC-derived progenies may have tumor potency in vivo [24, 46]. In a clinical setting, careful phenotyping of hiPSC-derived transplants will be necessary before implantation. To reduce the potential for tumorigenic outgrow of hiPSC-derived chondrocytes, we used the integration- and viral-free minicircle reprogramming technique. Although the reprogramming efficiency of this technique is relatively low, it prevents the potential reactivation of the pluripotency factors described for viral-derived hiPSCs and therefore lowers the risk of subsequent tumor formation [47].

In summary, we have developed a novel and clinically applicable approach for cartilage tissue regeneration via direct differentiation of patient-specific hiPSCs to hMSCs, without embryoid body formation. This approach could be widely used for more efficient and better controlled design of new cellular therapeutics for cartilage regeneration, thereby ultimately restoring articular cartilage function in patients with OA and decreasing associated morbidities. In the future, this technique could be applied for many other tissue regeneration applications, beyond our own focus on cartilage regeneration.
